# Albuminuria and neck circumference are determinate factors of successful accurate estimation of glomerular filtration rate in high cardiovascular risk patients

**DOI:** 10.1371/journal.pone.0185693

**Published:** 2018-02-02

**Authors:** Po-Jen Hsiao, Hung-Che Lin, Shih-Tai Chang, Jen-Te Hsu, Wei-Shiang Lin, Chang-Min Chung, Jung-Jung Chang, Kuo-Chun Hung, Yun-Wen Shih, Fu-Chi Chen, Fu-Kang Hu, Yi-Syuan Wu, Chi-Wen Chang, Sui-Lung Su, Chi-Ming Chu

**Affiliations:** 1 Division of Nephrology, Department of Internal Medicine, Tri-Service General Hospital, National Defense Medical Center, Taipei, Taiwan, R.O.C; 2 Division of Nephrology, Department of Internal Medicine, Taoyuan Armed Forces General Hospital, Taiwan, R.O.C; 3 Big Data Research Center, Fu-Jen Catholic University, Taiwan, R.O.C; 4 Department of Otolaryngology–Head and Neck Surgery, Tri-Service General Hospital, National Defense Medical Center, Taipei, Taiwan, R.O.C; 5 Division of Cardiology, Chang Gung Memorial Hospital-Linkou, Chang GungUniversity College of Medicine, Tao-Yuan, Taiwan, R.O.C; 6 Division of Cardiology, Department of Internal Medicine, Tri-Service General Hospital, National Defense Medical Center, Taipei, Taiwan, R.O.C; 7 School of Public Health, National Defense Medical Center, Taipei, Taiwan, R.O.C; 8 Department of Biomedical Engineering, National Defense Medical Center, Taipei, Taiwan, R.O.C; 9 School of Nursing, College of Medicine, Chang Gung University, Taoyuan, Taiwan, R.O.C; 10 Division of Endocrinology, Department of Pediatrics, Linkou Chang Gung Memorial Hospital, Taoyuan, Taiwan, R.O.C; 11 Graduate Institute of Life Sciences, National Defense Medical Center, Taipei, Taiwan, R.O.C; 12 Department of Public Health, China Medical University, Taichung City, Taiwan, R.O.C; Hospital Universitario de la Princesa, SPAIN

## Abstract

**Background:**

Estimated glomerular filtration rate (eGFR) is used for diagnosis of chronic kidney disease (CKD). The eGFR models based on serum creatinine or cystatin C are used more in clinical practice. Albuminuria and neck circumference are associated with CKD and may have correlations with eGFR.

**Aim:**

We explored the correlations and modelling formulates among various indicators such as serum creatinine, cystatin C, albuminuria, and neck circumference for eGFR.

**Design:**

Cross-sectional study.

**Methods:**

We reviewed the records of patients with high cardiovascular risk from 2010 to 2011 in Taiwan. 24-hour urine creatinine clearance was used as the standard. We utilized a decision tree to select for variables and adopted a stepwise regression method to generate five models. Model 1 was based on only serum creatinine and was adjusted for age and gender. Model 2 added serum cystatin C, models 3 and 4 added albuminuria and neck circumference, respectively. Model 5 simultaneously added both albuminuria and neck circumference.

**Results:**

Total 177 patients were recruited in this study. In model 1, the bias was 2.01 and its precision was 14.04. In model 2, the bias was reduced to 1.86 with a precision of 13.48. The bias of model 3 was 1.49 with a precision of 12.89, and the bias for model 4 was 1.74 with a precision of 12.97. In model 5, the bias could be lower to 1.40 with a precision of 12.53.

**Conclusions:**

In this study, the predicting ability of eGFR was improved after the addition of serum cystatin C compared to serum creatinine alone. The bias was more significantly reduced by the calculation of albuminuria. Furthermore, the model generated by combined albuminuria and neck circumference could provide the best eGFR predictions among these five eGFR models. Neck circumference can be investigated potentially in the further studies.

## Introduction

Over the past ten years, the prevalence and incidence of chronic kidney disease (CKD) and end-stage renal disease (ESRD) have risen rapidly in all countries, leading to further comorbiditity and mortality [1–3]. The glomerular filtration rate (GFR) is an indicator that is currently used to assess renal function. Currently, the gold standard for the confirmation of renal function is to inject exogenous substances such as inulin and nuclear medicinal substances into the body and to then detect the concentrations of these substances in the blood or urine to determine the glomerular filtration conditions [4]. Although these methods are highly accurate, the processes are complicated, time-consuming, and expensive. Moreover, clinically, the 24-hour urine creatinine clearance (24 hours CCR) is often used as a standard. However, this method is complicated and is easily affected by the degree of patient cooperation for urine collection [4].

Previously, the detection of renal function was often performed using methods such as collecting a single urine sample to measure trace proteins and collecting blood samples to measure serum creatinine levels. However, many studies have shown that determining kidney disease on the basis of urine or serum creatinine alone tends to overestimate renal function. In addition, serum creatinine is mainly affected by muscle mass. Thus, recently developed renal function assessment models have been established on the basis of endogenous indicators of serum creatinine and have been adjusted for factors that may affect creatinine, including age, gender, body surface area, and race, to enhance detection accuracy. Many studies have found that commonly used estimated GFR (eGFR) models, including the Modification of Diet in Renal Disease (MDRD) and Cockcroft-Gault (CG) model, demonstrate a poor predictive ability for the loss of kidney function at early stages. Moreover, particularly in Asian populations, these models are prone to overestimate renal function, such that patients with early CKD cannot be detected with accuracy [5,6]. Other single serum creatinine-based formula, The Chronic Kidney Disease Epidemiology Collaboration (CKD-EPI) equation has also been reported to be an accurate formula compared with MDRD. Despite serum creatinine, other endogenous substance such as cystatin C has been used more as an evaluation indicator. Serum cystatin C-based formula has been reported be more accurate than CKD-EPI equation [7–9]. Improved predicting ability of eGFR by combined serum creatinine and cystatin C measurements also has been reported [10–12].

In this clinical study, the GFR, which is reflected by 24 hours CCR corrected for body surface area, was used as the standard to assess renal function. Albuminuria is an important marker of estimation of kidney functions, which could help the detection of progressive CKD at early stages [13]. We measure the levels of albumin in urine as to assess for kidney damage, and compare the correlations of the concentrations of the endogenous serum indicators, creatinine and cystatin C, with changes in renal function. Additionally, neck circumference (NC) is an anthropometric measure of obesity for subcutaneous adipose tissue distribution which has been reported to be associated with cardiometabolic risk and CKD [14,15]. To date, the most used method for eGFR is still MDRD model in Taiwan. However, it is unsatisfied in clinical practice. We investigate whether albuminuria and NC is associated with renal function in high risk CKD patients. Finally, we establish five different models to calculate the eGFR and compare these traditional and more commonly used models in Taiwan.

## Methods

### Patients and data collection

#### Research framework

This study is a cross-sectional study, which analyzed the collected data and established models. This study was approved by the human trial committee of the Tri-Service General Hospital and Chiayi Chang Gung Memorial Hospital. Approval for this study was provided by the Institutional Review Board (IRB) of the Chang Gung Memorial Hospital (Approval No: CGMH- IRB-99-3623B).

#### Research subjects and data collection

The patient cases consisted of outpatients recruited from the cardiology clinics at the Tri-Service General Hospital and Chiayi Chang Gung Memorial Hospital from 2010–2011. The inclusion criteria included patients who were over 20 years of age, had normal renal function or were diagnosed between stages 1 to 5 with CKD. The exclusion criteria included patients with acute renal failure, hereditary kidney disease, other kidney associated diseases, cancer, pregnancy, breastfeeding, long-term use of steroids, or recent radioactive examinations. Patients who participated in the study were required to sign a consent form after we had explained the purpose and content of the study. In this present study, according to Magnani (1997) suggested the formulation for calculating sample size under 95% confidence interval which is alpha error as the following:

$$n=\frac{Z^{2}*pq}{m^{2}}$$

The sample size was determined around 139 = (1.96)^2×(0.9)×(0.1) / (0.05)^2.

### Outcome measures and definitions

#### Assessment of renal function

The GFR, which was represented by the 24 hours CCR adjusted for body surface area (rGFR) (ml/min/1.73 m^2^), was used as the standard for renal function assessment, and the urine albuminuria conditions were measured to assess for renal injury. The 24 hours CCR model was as follows: (24-hour urine volume × urine creatinine concentration) / (serum creatinine concentration × 1440), adjusted for a body surface area of 1.73 m^2^. To determine the level of albuminuria, the urinary albumin concentration was multiplied by the urine volume to obtain the 24-hour albuminuria concentration (mg/day). The creatinine concentration measurement was based on the Modified Jaffe Method and was determined using the SYNCHRON LXI 725 (Tokyo, Japan). The serum cystatin C concentration measurement method was based on the particle-enhanced turbidimetric immunoassay and performed using the Hitachi 7170 series analyzer (Tokyo, Japan).

### Statistical analysis

#### Data processing

For statistical analyses, SPSS 18.0 was used. If the data did not have a normal distribution, then the logarithmic transformation was used to normalize the data. Spearman’s correlation was used to analyze the correlations of the variables to examine the correlations of the serum biochemical parameters with the standard rGFR and kidney damage. The α-level was set at 0.05, and a p-value <0.05 indicated that the correlation between the variables was statistically significant.

#### Feature selection

In this present study, we analyzed the relations with measures of renal function among NC (which is much easier measured and less variance) and most anthropometric and renal physiological measurements such as body mass index, circumferences of waist and hip, age, systolic blood pressure, diastolic blood pressure, fasting blood glucose, serum triglyceride, high-density lipoprotein (HDL), low-density lipoprotein (LDL), total cholesterol, creatinine, cystatin C, blood urea nitrogen, uric acid, total protein, albumin, C-reactive protein, Glutamic Oxaloacetic Transaminase (GOT), Glutamic Pyruvic Transaminase (GPT), total bilirubin, calcium, phosphate, sodium, and microalbuminuria. Furthermore, we analyzed feature selection for modeling using the decision tree, including several variables (age, creatinine, cystatin C, NC, and microalbuminemia) (S1 Fig). The model equations included age, creatinine, cystatin C, NC, microalbuminemia, and gender for further regression models of eGFR compared with equations of CG and MDRD.

#### Model establishment

A decision tree was used to analyze and determine the variables of the model, and the basic case information, medical history, lifestyle, and blood biochemical indicator information were used as independent variables. The rGFR was used as the dependent variable. The results obtained using the decision tree were compared with Spearman’s correlation to determine the variables of the model. Stepwise multiple regression was performed using the selected variables as independent variables, and the logarithmically transformed eGFR was used as the dependent variable. Non-normal variables were also logarithmically transformed and placed into the variables to establish the eGFR model. Finally, the best-fit model was selected based on the R^2^ explanatory power of the model. The samples of this study were then placed back into the model to make predictions. In addition, the samples were also fitted into the MDRD and CG models to obtain the predictive values for each model, and the reliability and validity of the models were then compared.

## Results

### Study population characteristics

#### Basic demographic data

This study included a total of 177 outpatients who were recruited from cardiology clinics. The basic patient demographic data are shown in Table 1. The average age of the patients was 66.6 ± 9.6 years, and the majority of the patients were male (70.6%). The average neck circumference was 38.7 ± 3.8 cm. The average blood pressure was 135.7 ± 13.8/77.6 ± 8.2 mm Hg.

**Table 1:** Study characteristics of patients.

	Mean (SD)	Median (Q1, Q3)	min, max
Gender, n (%)			
Male	125 (70.6)		
Female	52 (29.4)		
Age (years)	66.6 (9.6)	65.5 (60.0, 74.2)	42.0, 88.1
Height (cm)	162.4 (7.3)	164.0 (158.0, 167.0),	143.0, 183.0
Weight (kg)	70.8 (12.1)	71.0 (62.0, 78.0)	47.0, 113.0
BMI (kg/m^2^)	26.8 (3.7)	26.6 (24.2, 28.7)	18.5, 40.9
Waist circumference (cm)	95.3 (9.8)	95.5 (90.0, 101.0)	71.0, 136.0
Neck circumference (cm)	38.7 (3.8)	38.0 (36.5, 41.0)	31.0, 49.0
Blood pressure (mm Hg)			
Systolic blood pressure	135.8 (13.8)	135.0 (126.3, 143.3)	88.0, 197.0
Diastolic blood pressure	77.6 (8.2)	77.7 (72.3, 82.3)	52.0, 103.0
Creatinine (mg/dL)	1.15 (0.44)	1.08 (0.92, 1.27)	0.58, 4.17
Cystatin C (mg/L)	1.05 (0.33)	0.96 (0.86, 1.15)	0.57, 2.84
Blood urea nitrogen (mg/dL)	18.7 (7.2)	17.9 (14.3, 20.6)	9.5, 60.4
Uric acid (mg/dL)	6.7 (1.6)	6.6 (5.7, 7.7)	2.5, 11.8
Albuminuria (mg/day)	174.2 (800.5)	11.8 (6.5, 40.0)	1.4, 8991.0
Normal (< 30 mg/day)	118 (68.6)		
Microalbuminuria (30–300 mg/day)	38 (22.1)		
Massive albuminuria (> 300 mg/day)	16 (9.3)		
rGFR (ml/min/1.73 m^2^)	50.4 (19.7)	49.2 (38.2, 62.6)	10.2, 112.2
≧90 ml/min/1.73 m^2^	5 (2.8)		
60–90 ml/min/1.73 m^2^	49 (27.7)		
45–60 ml/min/1.73 m^2^	46 (26.7)		
30–45 ml/min/1.73 m^2^	45 (26.2)		
15–30 ml/min/1.73 m^2^	21 (11.9)		
<15 ml/min/1.73 m^2^	6 (3.4)		
GFR_MDRD ml/min/1.73 m^2^	68.6 (19.3)	70.4 (56.8, 79.8)	14.7, 137.6
GFR_CG ml/min/1.73 m^2^	66.01 (24.1)	62.7 (50.7, 79.9)	10.5, 148.9

SD: standard deviation; BMI: body mass index; rGFR: Standard value of GFR; MDRD: Modification in Renal Disease; CG: Cockcroft-Gault

#### Blood and urine biochemical parameters

The average value of the GFR standard rGFR was 50.4 ± 19.7 ml/min/1.73 m^2^. After the creatinine level was fitted into the MDRD and CG models and converted, the eGFR values were 68.6 ± 19.3 ml/min/1.73 m^2^ and 66.6 ± 24.1 ml/min/1.73 m^2^, respectively. Compared with the standard rGFR, these two values showed a trend of overestimating the GFR. The sample average 24-hour albumin concentration was 176.1 ± 805.1 mg/day. If the cases were grouped according to the criterion of microalbuminuria, then 118 patients (68.6%) were considered normal (Table 1).

### Establishment of the renal function assessment model

The five models established in this study are shown in Table 2. We compared the eGFR calculated based on the five models with the gold standard value, and also fitted the data into the Cockcroft-Gaultc and MDRD models for comparison. Model 1 only considered serum creatinine with an adjustment for age and sex, where the explanatory power R^2^ was 0.522, and the mean predicted value was 48.35 ± 14.70 ml/min/1.73 m^2^. Model 2 added serum cystatin C into the model, where the explanatory power was increased to 0.551, and the mean predicted value was 48.47 ± 14.70 ml/min/1.73 m^2^. In model 3, albuminuria was added, where the explanatory power was increased to 0.622, and the mean predicted value was 48.84 ± 16.04 ml/min/1.73 m^2^. In addition to the original age, gender, serum creatinine, and cystatin C, model 4 also added NC, where the explanatory power was 0.576, and the mean predicted value was 48.59 ± 14.99 ml/min/1.73 m^2^. Model 5 added six variables into the model, where the explanatory power was increased to 0.635, and the mean predicted value was 48.90 ± 16.28 ml/min/1.73 m^2^.

**Table 2:** Comparison of the error values, precision and relevance of each GFR equation.

	rGFR	MDRD	CG	Equation 1	Equation 2	Equation 3	Equation 4	Equation 5
Mean	50.33	68.54	66.22	48.35	48.47	48.84	48.59	48.90
Standard deviation	19.26	19.66	24.01	14.70	14.54	16.04	14.99	16.28
bias		-18.21	-15.89	2.01	1.86	1.49	1.74	1.4
p-value		<0.001	<0.001	0.062	0.093	0.159	0.104	0.168
precision		16.28	16.95	14.04	13.48	12.89	12.97	12.53
accuracy								
within 30%		-	-	43.6%	42.3%	36.2%	39.6%	31.5%
within 50%		-	-	59.3%	61.7%	53.7%	63.8%	54.4%
within 70%		-	-	79.9%	76.5%	77.2%	80.5%	77.9%
R^2^		-	-	0.522	0.551	0.622	0.576	0.635
r		0.65	0.714	0.702	0.715	0.748	0.74	0.764
p-value		<0.001	<0.001	<0.001	<0.001	<0.001	<0.001	<0.001

Bias: mean difference; precision: SD of bias; CG: Cockcroft-Gault; crea: creatinine; cysC: cystatin C; NC: neck circumference; microalb: microalbuminuria

Cockcroft-Gault: ((140-age)×weight)/(0.72×crea)×(0.85 if female)

MDRD: 186× crea^-1.154^ ×age^-0.203^ × (0.742 if female)×(1.212 if black)

Equation 1: 2387 ×age^-0.894^ ×crea^-0.998^× (0.100 if female)

Equation 2: 1504 ×age^-0.731^ ×crea^-0.819^ ×cysC^-0.235^× (0.162 if female)

Equation 3: 554 ×age^-0.610^ ×crea^-0.903^ ×cysC^-0.414^×microalb^0.082^ × (0.704 if female)

Equation 4: 23 ×age^-0.625^ ×crea^-0.784^ ×cysC^-0.299^ ×NC^0.947^ (0.502 if female)

Equation 5: 24 ×age^-0.495^ ×crea^-0.871^ ×cysC^-0.45^ ×NC^0.45^× microalb^0.077^× (0.502 if female)

We compared the reliability and validity of each model in predicting the GFR. The mean rGFR was 50.33 ± 19.26 ml/min/1.73 m^2^, and the MDRD and Cockcroft-Gaultc models, which were established based on serum creatinine alone, yielded mean values of 68.54 ± 19.66 ml/min/1.73 m^2^ and 66.22 ± 24.01 ml/min/1.73 m^2^, respectively (Table 2). Biases of the two models compared with the mean rGFR were -18.21 and -15.89 with a precision of 16.28 and 16.95, respectively, and the both biases showed significant differences. Model 1 was established based on serum creatinine alone, where its bias was 2.01 and its precision was 14.04. The p-value for the bias between the two was 0.062. After the addition of serum cystatin C to model 2, the bias between the predicted value and the rGFR was reduced to 1.86 with a precision of 13.48, indicating that after the addition of cystatin C, the predicting ability was improved compared to the models for creatinine alone. Furthermore, models 3 and 4 added albuminuria and NC, respectively. The bias of model 3 was 1.49 with a precision of 12.89, and the bias for model 4 was 1.74 with a precision of 12.97. These results suggested that the bias was more significantly reduced by the addition of albuminuria. Model 5 simultaneously added both variables, and the bias was 1.40 with a precision of 12.53. The correlation of serum cystatin C with rGFR was higher compared to creatinine, while the difference in the correlation with albumin concentration between the two indicators was only visible in cases of massive albuminuria (r = 0.785 vs. r = 0.597) (Table 3).

**Table 3:** Comparison of the correlations of the biochemical parameters of creatinine and cystatin C with rGFR and albuminuria after stratification on the basis of the severity of albuminuria.

	Normal albuminuria (n = 118) <30 mg/day				Microalbuminuria (n = 38) 30–300 mg/day				Massive albuminuria (n = 16) >300 mg/day			
	rGFR		albumin concentration		rGFR		albumin concentration		rGFR		albumin concentration	
	coefficient	p-value	coefficient	p-value	coefficient	p-value	coefficient	p-value	coefficient	p-value	coefficient	p-value
creatinine	-0.328	<0.001	0.038	0.686	-0.690	<0.001	0.011	0.948	-0.874	<0.001	0.597	0.005
cystatin C	-0.422	<0.001	0.015	0.878	-0.695	<0.001	0.227	0.212	-0.897	<0.001	0.785	0.001

rGFR: Standard value of GFR

## Discussion

### Relationship between serum creatinine and renal function and between serum cystatin C and renal function

Patients with early stage CKD may simultaneously exhibit glomerular filtration abnormalities and abnormal albuminuria caused by kidney damage, but may also only display decreased glomerular filtration or pathological lesions in the kidneys [16]. Several studies have shown that serum cystatin C responds more sensitively to a reduction in GFR and that serum cystatin C may replace creatinine as an indicator to measure renal function. In addition, the sensitivity of the serum cystatin C response to decreased GFR is higher compared to serum creatinine because creatinine is re-secreted by the renal tubules. However, the creatinine response in early stages of glomerular decline is unclear, and creatinine only increases when the deterioration of renal function enters stage 3 [4,17]. Studies on diabetic patients or healthy subjects have found that serum cystatin C is more sensitive during the early stages of renal dysfunction [5,18]. Compared with serum creatinine, some studies also recommend that serum cystatin C-based eGFR had good clinical utility in specific populations [19–22]. However, other studies have indicated that although serum cystatin C demonstrates a highly sensitive response to decreased GFR at early stages of impaired renal function, the correlation of these concentration changes with renal function is decreased when renal function deteriorates in the late stages, whereas the sensitivity of serum creatinine increases at late stages [12].

Albuminuria is an important marker of estimation of kidney functions, which can help the detection of progressive CKD at early stages [13]. Therefore, our study further divided the patients into different groups on the basis of the degree of kidney damage, and investigated the relationships between the indicators and rGFR, as well as that between the indicators and albumin concentration. The correlation of serum cystatin C with rGFR was obvious higher compared to creatinine. The difference in the correlation with albumin concentration between the two indicators was observed in cases of massive albuminuria (Table 3). These findings suggested that the rGFR conditions as determined by serum cystatin C might also reflect conditions of albuminuria. The changes in the concentration of serum cystatin C may also reflect the GFR condition. Previous study demonstrated that serum cystatin C and GFR were not significantly correlated in patients with normal levels of albuminuria, suggesting the correlation between serum cystatin C and renal function was due to an interaction between cystatin C and albuminuria, rather than a reflection of a decline in GFR [23].

### Model establishment and comparison

Previous assessment of renal function is often determined based on urine or blood creatinine values alone. However, because creatinine is a substance produced in muscle metabolism, individual differences may affect the concentration of creatinine, and it may not be appropriate to use this indicator alone to determine renal function. However, the Cockcroft-Gault and MDRD models, which are commonly used internationally, were established solely based on serum creatinine [4]. In contrast, this study attempted to add serum cystatin C, albuminuria, and NC into the eGFR model, to improve the ability to predict renal function.

This study established five eGFR models. Model 1 was based on only serum creatinine and was adjusted for age and gender. A comparison of model 1 and the Cockcroft-Gault and MDRD models with rGFR revealed that the Cockcroft-Gault and MDRD models had a relatively large bias compared with rGFR, which was consistent with other studies. The level of creatinine may also differ according to race, but the Cockcroft-Gault model does not adjust for race and the MDRD model, only distinguishing between blacks and whites. Thus, many studies have shown that these models demonstrate a poor predictive ability for early stages of kidney disease. Furthermore, particularly for Asians, the extent of the underestimate is more robust [4,6]. Model 2 added serum cystatin C, where the explanatory power showed a slight increase compared to model 1. Furthermore, the bias between the predictive value of eGFR and rGFR decreased from 2.01 to 1.86, and the precision also showed a slight reduction. These findings suggested that after adding serum cystatin C, the bias of the predicted value compared to rGFR will decrease. In addition, models 3 to 5 sequentially added albuminuria and NC. The bias had decreased significantly after adding albuminuria, and the explanatory power also increased to 0.62. Although the explanatory power had only increased to 0.58 after the addition of NC, the accuracy rates within 50% and 70% were increased to 63.8% and 80.5%, respectively, which was more accurate compared to the other models. NC is an easy associated method for metabolic syndrome and insulin resistance and reported to be a powerful indicator of atherosclerotic lipid abnormalities and their risk factors recently [24–26]. In our study, the correlations of NC with rGFR (ml/min/1.73m2) and microalbuminuria (mg/day) were 0.382 (p<0.001) and 0.304 (p<0.001), respectively (S1 Table). Moreover, the correlations of NC with body mass index (BMI) (kg/m^2^), waist circumference (cm), and hip circumference (cm) were 0.566 (p<0.001), 0.515 (p<0.001), and 0.626 (p<0.001), respectively (S2 Table). The analysis revealed that NC was not only much easier measured and less variance, but also a good indicator for renal function and high correlations with BMI (kg/m^2^), waist circumference (cm), and hip circumference (cm).

Other studies focusing on model adjustment found that models established based on serum creatinine alone often demonstrated less stable bias, lower precision and lower validity, and the 30% accuracy was approximately 53% to 80%. After the inclusion of serum cystatin C, the stability increased to approximately 87%, and the explanatory power was between 70% to 95% [11,12,27]. However, the 30% accuracy obtained in our study was approximately within the range of 31.5% to 43.6%, and 50% and 70% accuracy was within the range of 53.7% to 63.8% and 77.2% to 80.5%, respectively, with an explanatory power range from 0.52% to 0.64%, which was slightly lower compared to other studies. This might be due to the different measurement methods for the gold standard values used to assess renal function. These studies often used the clearance of nuclear medicinal substances to detect renal function, and their detection stability and accuracy were more accurate than the GFR estimated-based method on 24 hours CCR.

This study has several limitations. First, a new equation always shows best performance in the development big data-set. The sample size in this study is relatively small and the study is done only on Taiwan patients. We do not know whether our equations could also be applied to patients from other ethnic backgrounds. This is the major limitation. Use a validation data-set to compare a new equation with previous one may be investigated in the future. Second, this study utilized the renal function gold standard of 24 hours CCR, which is one of the gold standards used in clinical practice to assess renal function. This method requires the collection of 24-hour urine samples from individual patients. Although it is not invasive, it is very time consuming, and the accuracy is dependent on the cooperation of individual patients. For example, patients might miss the collection during the process of urine collection or use incorrect storage methods. These factors are likely to result in an incorrect assessment of renal function. Thus, when collecting data for this study, we printed specific 24-hour urine collection procedures and precautions to reduce the incidence of bias and to improve detection accuracy. Furthermore, 24 hours CCR is usually greater than GFR by tubular secretion of creatinine. This may be another limitation in this study. Finally, this is a cross-sectional study and do not infer the causal relationships between the indicators and disease. CKD staging also requires further verification by a long-term follow-up to determine whether the decline in renal function continued for over three months and whether the abnormal conditions of albuminuria continued to occur within one week.

## Conclusions

Our study results demonstrated the predicting ability of eGFR got improvement after the addition of serum cystatin C compared to the traditional models for serum creatinine alone. And the bias could be more significantly reduced by the calculation of albuminuria. The equations combined albuminuria and NC could help provide more accurate eGFR predictions. Anthropometric measures of obesity for subcutaneous adipose tissue distribution, such as NC can also be investigated more in the future.

## Supporting information

S1 Fig Decision tree flow of this study. (DOCX)

S1 Table Correlation of clinical and biochemical variables with rGFR and microalbuminuria. (DOCX)

S2 Table Correlation of clinical and biochemical variables with neck circumference. (DOCX)
