# Supplementary figures and images for: Albuminuria and neck circumference are determinate factors of successful accurate estimation of glomerular filtration rate in high cardiovascular risk patients
Source: PLoS One. 2018 Feb 2;13(2):e0185693. doi: 10.1371/journal.pone.0185693 (PMC5796684; doi:10.1371/journal.pone.0185693)

**S1 Fig. Decision tree flow of this study.**


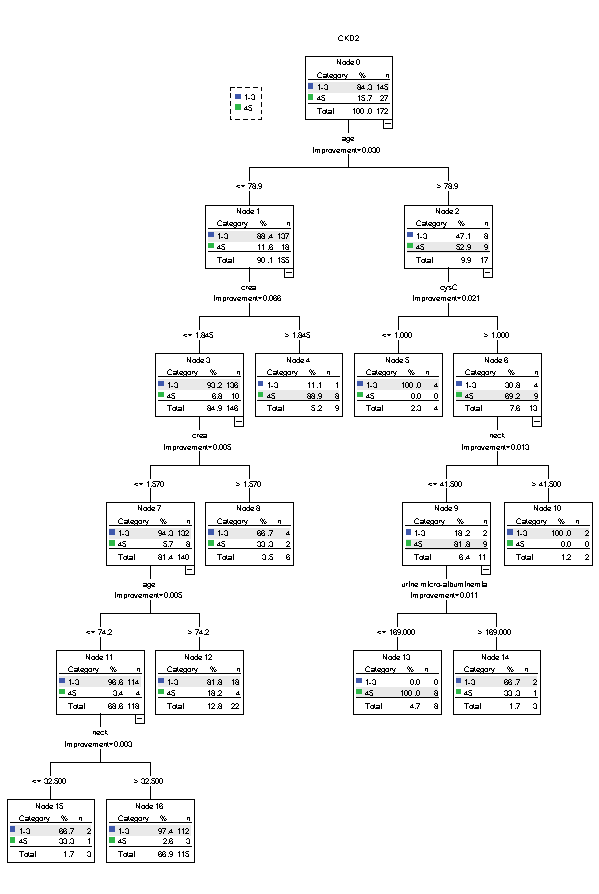

Supplement: S1 Fig — (DOCX) [file pone.0185693.s001.docx]
