# Supplementary material for: Albuminuria and neck circumference are determinate factors of successful accurate estimation of glomerular filtration rate in high cardiovascular risk patients
Source: PLoS One. 2018 Feb 2;13(2):e0185693. doi: 10.1371/journal.pone.0185693 (PMC5796684; doi:10.1371/journal.pone.0185693)
Supplement: S1 Table — (DOCX) [file pone.0185693.s002.docx]

**S1 Table. Correlation of clinical and biochemical variables with rGFR and microalbuminuria.**

|  |  | |  | |
| --- | --- | --- | --- | --- |
|  | **rGFR**  (ml/min/1.73 m^2^) | | **Microalbuminuria** (mg/day) | |
|  | **correlation** | **p-value** | **correlation** | **p-value** |
| Age (year) | -0.461*** | <0.001 | -0.049 | 0.526 |
| BMI (kg/m^2^) | 0.327*** | <0.001 | 0.312*** | <0.001 |
| Waist circumference (cm) | 0.195** | 0.01 | 0.284*** | <0.001 |
| Hip circumference (cm) | 0.368*** | <0.001 | 0.246** | 0.001 |
| Neck circumference (cm) | 0.382*** | <0.001 | 0.304*** | <0.001 |
| Systolic blood pressure (mmHg) | 0.084 | 0.274 | 0.300*** | <0.001 |
| Diastolic blood pressure (mmHg) | 0.196** | 0.01 | 0.091 | 0.238 |
| Fasting blood glucose (mg/dL) | 0.008 | 0.913 | 0.142 | 0.063 |
| Triglyceride (mg/dL) | 0.027 | 0.724 | 0.133 | 0.181 |
| HDL (mg/dL) | -0.036 | 0.645 | -0.250** | 0.001 |
| LDL (mg/dL) | 0.089 | 0.247 | -0.056 | 0.462 |
| Total chrolesterol (mg/dL) | 0.043 | 0.577 | -0.107 | 0.164 |
| Creatinine (mg/dL) | -0.444*** | <0.001 | 0.221** | 0.004 |
| Cystatin C (mg/L) | -0.51*** | <0.001 | 0.256** | 0.002 |
| BUN (mg/dL) | -0.288*** | <0.001 | 0.232** | 0.003 |
| Uric acid (mg/dL) | -0.143 | 0.063 | 0.267*** | <0.001 |
| Albumin (mg/dL) | 0.267*** | <0.001 | -0.048 | 0.530 |
| C-reactive protein (mg/dL) | -0.048 | 0.536 | 0.225** | 0.003 |
| Microalbuminemia (mg/dL) | -0.027 | 0.729 | - | - |
| Total protein (mg/dL) | -0.009 | 0.908 | 0.089 | 0.248 |
| GOT (mg/dL) | -0.081 | 0.298 | 0.074 | 0.340 |
| GPT (mg/dL) | 0.169* | 0.026 | 0.166* | 0.030 |
| Total bilirubin (mg/dL) | 0.175* | 0.023 | -0.012 | 0.872 |
| Calcium (mg/dL) | 0.051 | 0.506 | -0.076 | 0.325 |
| Phosphate (mmol/L) | -0.023 | 0.766 | -0.008 | 0.915 |
| Sodium (mmol/L) | 0.108 | 0.161 | -0.094 | 0.224 |

*p<0.05；**P<0.01；***p<0.001
