# Supplementary material for: Albuminuria and neck circumference are determinate factors of successful accurate estimation of glomerular filtration rate in high cardiovascular risk patients
Source: PLoS One. 2018 Feb 2;13(2):e0185693. doi: 10.1371/journal.pone.0185693 (PMC5796684; doi:10.1371/journal.pone.0185693)
Supplement: S2 Table — (DOCX) [file pone.0185693.s003.docx]

**S2 Table. Correlation of clinical and biochemical variables with neck circumference.**

|  | **Neck circumference** (cm) | |
| --- | --- | --- |
|  | **correlation** | **p-value** |
| rGFR (ml/min/1.73 m^2^) | 0.382*** | <0.001 |
| Albumin (mg/dL) | 0.304*** | <0.001 |
| BMI (kg/m^2^) | 0.566*** | <0.001 |
| Waist circumference (cm) | 0.515*** | <0.001 |
| Hip circumference (cm) | 0.626*** | <0.001 |
| Systolic blood pressure (mmHg) | 0.180* | 0.017 |
| Diastolic blood pressure (mmHg) | 0.278*** | <0.001 |
| Fasting blood glucose (mg/dL) | 0.161* | 0.035 |
| Triglyceride (mg/dL) | 0.208** | 0.006 |
| HDL (mg/dL) | -0.263** | 0.001 |
| LDL (mg/dL) | 0.100 | 0.192 |
| Total chrolesterol (mg/dL) | -0.071 | 0.355 |
| Creatinine (mg/dL) | 0.181* | 0.017 |
| Cystatin C (mg/L) | 0.104 | 0.209 |
| BUN (mg/dL) | 0.120 | 0.126 |
| Uric acid (mg/dL) | 0.191* | 0.012 |
| Albumin (mg/dL) | 0.167* | 0.030 |
| C-reactive protein (mg/dL) | 0.161* | 0.036 |
| Total protein (mg/dL) | -0.032 | 0.677 |
| GOT (mg/dL) | -0.0.26 | 0.734 |
| GPT (mg/dL) | 0.156* | 0.040 |
| Total bilirubin (mg/dL) | 0.075* | 0.330 |
| Calcium (mg/dL) | 0.070 | 0.366 |
| Phosphate (mmol/L) | -0.274 | <0.001 |
| Sodium (mmol/L) | -0.042 | 0.589 |

*p<0.05；**P<0.01；***p<0.001
